# Management of recurrent intussusception after Roux-en-Y gastric bypass

**DOI:** 10.1093/jscr/rjae778

**Published:** 2024-12-19

**Authors:** Ankur Makani, Andrew Hendrix, Gunnar Orcutt, Christopher Stephenson, Thomas Crafton, David Moffatt

**Affiliations:** Prisma Health Department of General Surgery, Columbia, SC 29205, United States; University of South Carolina School of Medicine, Columbia, SC 29205, United States; Prisma Health Department of General Surgery, Columbia, SC 29205, United States; Prisma Health Department of General Surgery, Columbia, SC 29205, United States; Prisma Health Department of General Surgery, Columbia, SC 29205, United States; Prisma Health Department of General Surgery, Columbia, SC 29205, United States

## Abstract

While intussusception is a well described complication of Roux-en-Y gastric bypass (RYGB), cases of recurrent intussusception after lead point resection and reconstruction are described much less frequently. We present a case of a 28-year-old female with triple recurrent intussusception all of which were treated with surgical resection and reconstruction of her RYGB anastomoses. There is currently limited evidence to direct management in the setting of recurrent intussusception. This case highlights the importance of considering intussusception in the RYGB patient with abdominal pain as well as demonstrates a need for further investigation into pathomechanisms which may lead to recurrence.

## Introduction

Evidence shows that bariatric and metabolic surgery is the most effective treatment for severe obesity and its clinical complications such as type 2 diabetes, hypertension, and heart disease [1]. Roux-en-Y gastric bypass (RYGB), first described by Mason in 1967, has become a prominent procedure in the realm of bariatric surgery [2]. Data from the 8th IFSO Global Registry Report show that RYGB makes up 28.5% of bariatric surgical interventions as of 2023, ranking second only to sleeve gastrectomy (62.5%) [3]. Common complications, such as chronic or recurrent abdominal pain, dumping syndrome, and post-bariatric hypoglycemia, necessitate hospital readmission for medical management in ⁓21.9% of cases within 4 years post-operatively [4].

Although rare in the adult population, intussusception comprises ⁓1%–5% of bowel obstructions. Adult intussusception typically signals an underlying pathologic condition, such as malignancy, polyps, colonic diverticulum, or strictures and necessitates surgical intervention [5]. The relationship between RYGB and intussusception is well documented. Studies have reported varying incidence rates, with a 2021 meta-analysis revealing a pooled incidence of 0.64% following RYGB with a median interval of 52 months between RYGB and intussusception occurrence [6]. Risk factors associated with post-RYGB intussusception include female sex (98%) as well as a significant postoperative weight loss described as ˃65% of excess weight [6]. Additionally, it is important to note that while the majority of intussusception cases within the adult population are anterograde (proximal telescopes into distal), retrograde intussusception (RI) is more frequent in RYGB patients [7]. Complications stemming from recurrent intussusception encompass obstruction, ischemia, necrosis, and infection, necessitating prompt diagnosis via clinical assessment and computed tomography scans, often followed by surgical exploration due to the elevated risk of bowel ischemia.

## Case presentation

**Figure 1:**
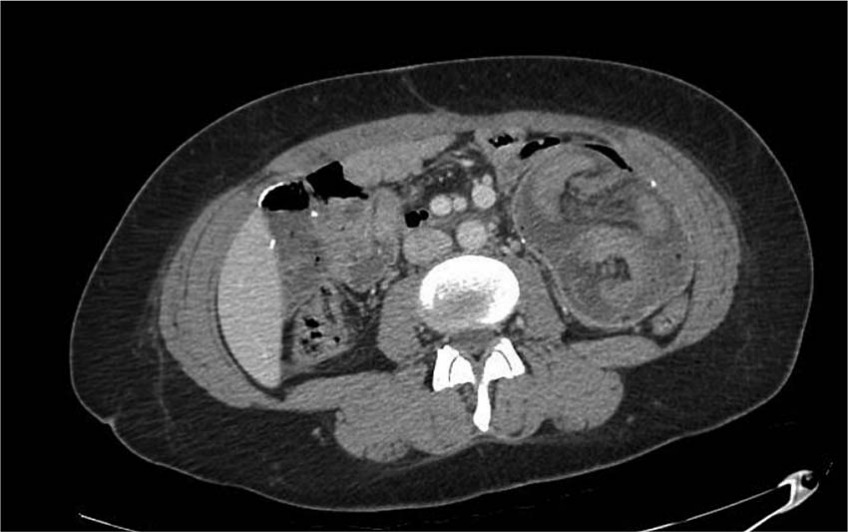
CT of the jejunojejunostomy intussusception.

**Figure 2:**
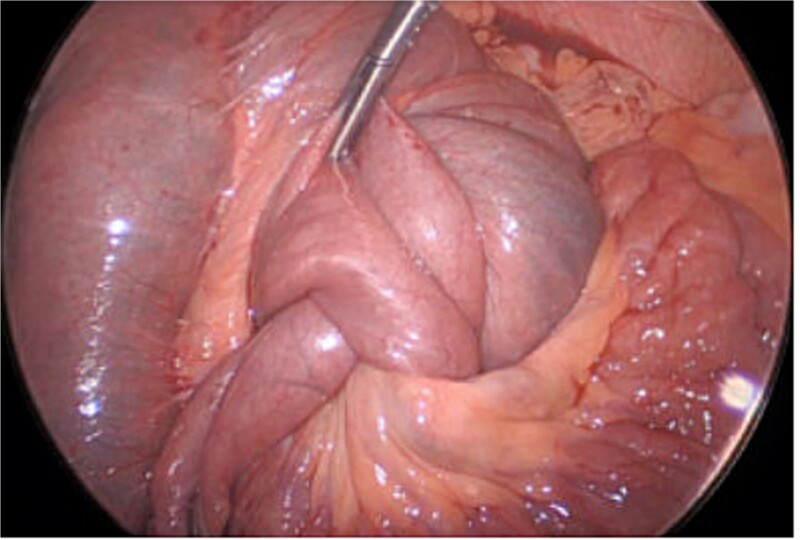
Intraoperative view of the jejunojejunostomy intussusception.

A 28-year-old female with a past medical history of morbid obesity and recurrent intussusception treated with laparoscopic RYGB, laparoscopic revision of jejunojejunostomy, and revision of proximal roux limb reconstituting anastomosis, presented to the emergency department with severe, unremitting abdominal pain. Her index RYGB was performed 6 years prior to presentation and most recent exploratory laparotomy and reconstruction of bypass anatomy occurred ⁓1 year prior. Four years following her RYGB, the patient presented for acute abdominal pain and was found to have intussusception of the jejunojejunostomy necessitating laparoscopic excision of the affected segment with reconstruction of bypass anatomy. Four months after her initial revision, she presented with acute abdominal pain and was found to have a second recurrence of intussusception and required a second laparoscopic excision of jejunojejunostomy with reconstruction of bypass anatomy. On this presentation, physical exam findings were consistent with peritonitis and computed tomography (CT) of her abdomen and pelvis revealed small bowel intussusception in the left abdomen measuring up to 9.6 × 9.1 × 14.7 cm in diameter with decreased enhancement of the small bowel involved in the intussusception concerning for ischemia (Fig. 1). We emergently proceeded to the operating room for diagnostic laparoscopy. On initial investigation, the roux limb reconstituting anastomosis was noted to be healthy appearing; however, there was a long segment of RI of the proximal common channel into the jejunojejunostomy with an obstructed biliopancreatic limb (Fig. 2). This was not reducible and we converted to an open approach for resection and revision of the jejunojejunostomy with reconstruction of the Roux-en-Y anatomy via a stapled, side-to-side antiperistaltic anastomosis between the distal Roux limb and proximal common channel. The patient tolerated the procedure well and has progressed well in the outpatient setting with close monitoring and follow-up.

## Discussion

While the pathophysiology underlying this complication has yet to be described, several mechanisms have been postulated. One theory involves the anastomotic staple line acting as a lead point [8]. Another theory focuses on alterations in small bowel motility induced by the surgical procedure [8]. In RYGB, transection of the jejunum disrupts the duodenal pacemaker, potentially leading to ectopic pacemaker potentials within the Roux limb and facilitating retrograde telescoping into the jejunal anastomosis [8]. Furthermore, it is also believed that significant weight loss may lead to elongation and thinning of the mesentery, which in turn provides less resistance and a higher predisposition to invagination [6].

To the best of our knowledge this is the first documented case of triple recurrence intussusception in a RYGB despite resection and reconstruction of RYGB anastamoses. While initial intussusception incidence is roughly 0.64% following RYGB, meta-analyses, and systematic reviews have reported recurrence rates ranging from 7.7% to 22% during follow-up [6, 9]. This data demonstrates that while rare, initial intussusception increases one’s chances of recurrence. Management strategies commonly include reduction with or without resection and reconstruction of the jejunojejunal anastomosis. While there is currently limited evidence to guide management in RYGB patients experiencing recurrent intussusception, a recent meta-analysis found resection and reconstruction of the jejunojejunostomy appears to be associated with the lowest risk of recurrence [10]. Of note, a prospective study did investigate the efficacy of reversing the gastric bypass with subsequent sleeve gastrectomy but found an increase in complications such as splenic bleeding, gastric fistula, superior mesenteric vein thrombosis, and small bowel obstruction [10].

## Conclusion

As the frequency of bariatric surgical procedures continues to increase, it is important to consider intussusception and recurrent intussusception as a potential cause of abdominal pain and small bowel obstruction in RYGB patients despite previous surgical intervention involving resection and revision of anastomosis. The potentially life-threatening implications associated with recurrent intussusception in RYGB patients underscores the importance of further investigation into risk factors and pathophysiology to enhance preventive measures and refine surgical techniques.
